# Pre-clinical model of dysregulated FicD AMPylation causes diabetes by disrupting pancreatic endocrine homeostasis

**DOI:** 10.1016/j.molmet.2025.102120

**Published:** 2025-03-10

**Authors:** Amanda K. Casey, Nathan M. Stewart, Naqi Zaidi, Hillery F. Gray, Hazel A. Fields, Masahiro Sakurai, Carlos A. Pinzon-Arteaga, Bret M. Evers, Jun Wu, Kim Orth

**Affiliations:** 1 Department of Molecular Biology, University of Texas Southwestern Medical Center, Dallas, TX, 75390, USA; 2 Howard Hughes Medical Institute, Dallas, TX, 75390, USA; 3 Department of Pathology, University of Texas Southwestern Medical Center, Dallas, TX 75390, USA; 4 Cecil H. and Ida Green Center for Reproductive Biology Sciences, University of Texas Southwestern Medical Center, Dallas, TX 75390, USA; 5 USA; Hamon Center for Regenerative Science and Medicine, University of Texas Southwestern Medical Center, Dallas, TX 75390, USA; 6 Department of Biochemistry, University of Texas Southwestern Medical Center, Dallas, TX, 75390, USA

**Keywords:** AMPylation, BiP, FicD, Insulin, Islet biology, Neonatal diabetes, Unfolded protein response

## Abstract

The bi-functional enzyme FicD catalyzes AMPylation and deAMPylation of the endoplasmic reticulum chaperone BiP to modulate ER homeostasis and the unfolded protein response (UPR). Human hFicD with an arginine-to-serine mutation disrupts FicD deAMPylation activity resulting in severe neonatal diabetes. We generated the m*FicD*^*R371S*^ mutation in mice to create a pre-clinical murine model for neonatal diabetes. We observed elevated BiP AMPylation levels across multiple tissues and signature markers for diabetes including glucose intolerance and reduced serum insulin levels. While the pancreas of m*FicD*^*R371S*^ mice appeared normal at birth, adult *mFicD*^*R371S*^ mice displayed disturbed pancreatic islet organization that progressed with age. *mFicD*^*R371S*^ mice provide a preclinical mouse model for the study of UPR associated diabetes and demonstrate the essentiality of FicD for tissue resilience.

## Highlights

• *FicD*^*R371S*^ mutation recessively alters global AMPylation of BiP.
• Loss of BiP deAMPylation alters UPR signaling in the pancreas resulting in neonatal diabetes.
• *FicD*^*R371S*^ mutation leads to disruption of pancreatic islet organization and function.
• *FicD*^*R371S*^ mutation leads to disruption of pancreatic islets that progresses with age after birth.
• *FicD*^*R371S*^ mice provide a preclinical mouse model for UPR associated diabetes.

## Graphical abstract

**Figure:**
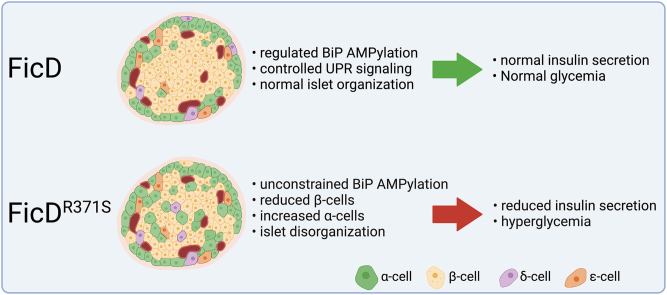

## 1 Introduction

The unfolded protein response (UPR) is a cellular stress response that plays a critical role in the regulation of protein homeostasis in the endoplasmic reticulum (ER). The UPR consists of signaling branches controlled by the three transducers: Inositol-requiring enzyme-1α (IRE1), protein kinase R-like endoplasmic reticulum kinase (PERK), and activating transcription factor 6 (ATF6). These three branches are activated when unfolded proteins accumulate in the lumen of the ER and bind to the ER Hsp70 chaperone protein BiP, resulting in the disassociation of BiP from the three transducers [[1], [2], [3]]. During an excess unfolded protein, cells can become stressed and induce the UPR, resulting in global inhibition of translation and increased production chaperones like BiP. If the stress is too great or prolonged, the UPR can signal for cells to die, and tissues can be damaged [4,5].

**Figure 1:**
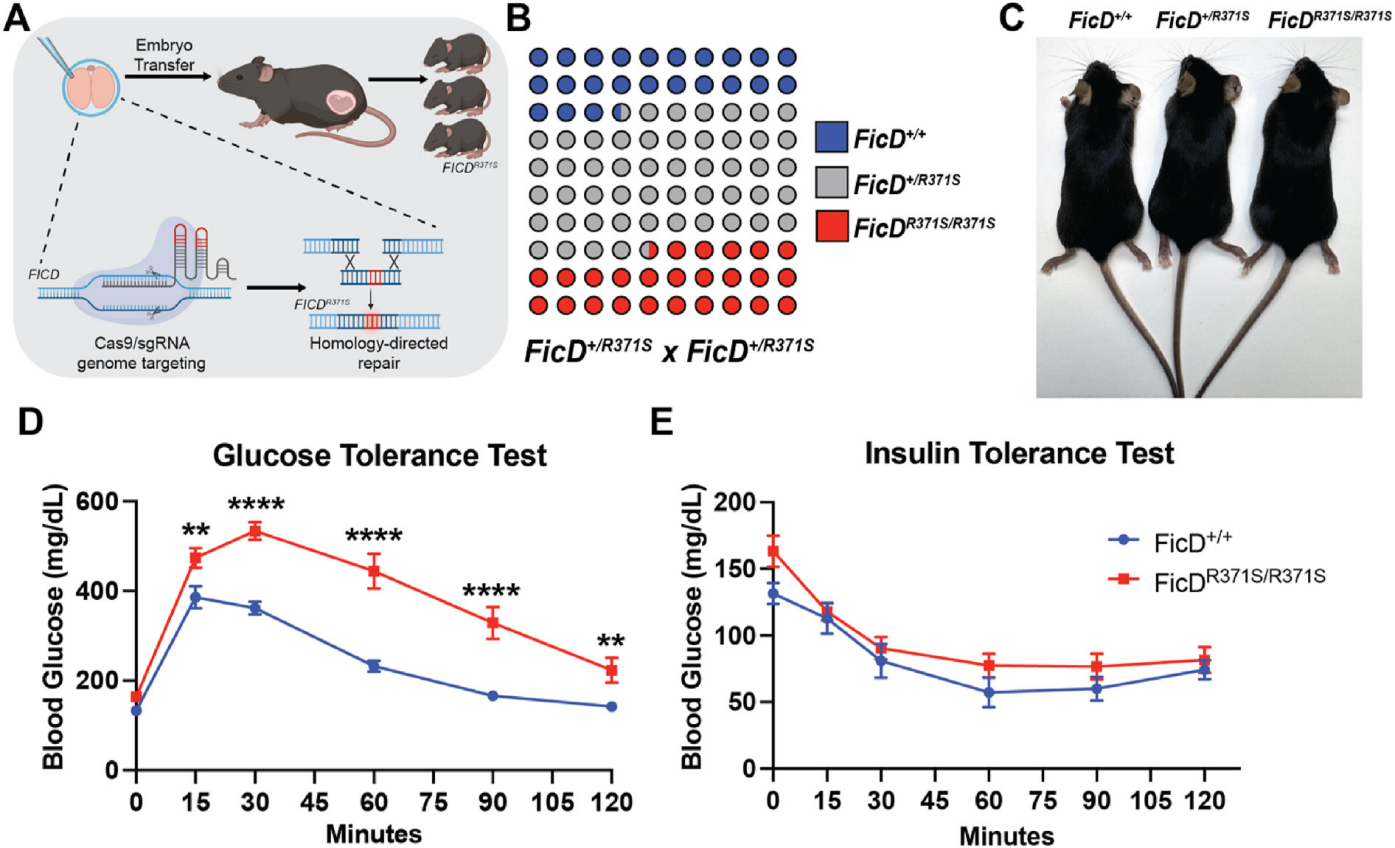
Generation of a neonatal diabetic FicD^R371S^ mouse model. (A) Diagram for the generation of *FicD*^*R371S*^ mice. Created in BioRender. Casey, A. (2025) https://BioRender.com/k62i549 (B) Schematic representing the distribution of genotypes from 14 FicD^*+/R371S*^ x *FicD*^*+/R371S*^ breeding cages, a total of 58 litters, 305 pups total. (C) Comparison of 10-week-old female *FicD*^*+/+*^, *FicD*^*+/R371S*^, and *FicD*^*R371S/R371S*^ littermates. (D) GTT and (E) ITT of 15 to 16-week-old male *FicD*^*+/+*^ (blue) and *FicD*^*R371S/R371S*^ (red) mice. Data indicate mean and error bars represent standard error. Statistics were performed using GraphPad Prism 10 using a 2-way ANOVA. N = 6. ∗∗, p < 0.01; ∗∗∗, p < 0.001; ∗∗∗∗, p < 0.0001.

In metazoans, the ER chaperone BiP is tempered by FicD, an ER-resident enzyme responsible for the reversible AMPylation of BiP in response to changes in ER stress [6]. Under conditions of low ER stress, FicD AMPylates BiP, thereby inactivating the chaperone and reducing its activity (Figure 1). During increased ER stress, FicD switches its function to deAMPylate BIP, rapidly increasing the pool of active chaperone to handle the elevated stress [7]. This dual catalytic activity of FicD is crucial for maintaining the appropriate level of active BiP during fluctuations of ER stress conditions. In previous studies with genetic models of FicD deletion in flies and mice, we and others have observed that the absence of FicD results in tissues being less resilient to physiological and environmental stress but not acute pharmacological stress [[8], [9], [10]].

Insulin synthesis and secretion is a highly complex process involving multiple steps in protein processing and trafficking. Throughout the lifetime of an organism, β-cells must constantly adapt to fluctuating insulin demands. While β-cells are generally resistant to transient ER stress, prolonged or chronic ER stress and UPR activation leads to impaired function and a loss of β-cell identity [11,12]. Prolonged activation of UPR pathways have been implicated in the progression of both type I and type II diabetes [[11], [12], [13]]. Furthermore, disruption of ER homeostasis and dysregulation of the UPR are key contributors to β-cell dysfunction in many monogenic syndromes of diabetes [14,15].

Recently, two recessive, disease-causing mutations in the human FicD gene have been discovered. *hFicD*^*R371S*^ was identified as a cause of infancy-onset diabetes and *hFicD*^*R34H*^ was shown to be associated with a progressive motor neuron disease [16,17]. These mutations disrupt hFicD's enzymatic activity, specifically impairing its ability to deAMPylate BiP in the ER [16,17]. In this study, we focused on the *hFicD*^*R371S*^ mutation to understand how this mutation impacts homeostasis and physiology of affected individuals and leads to neonatal diabetes. For this study, we developed a pre-clinical murine model carrying a recessive *mFicD*^*R371S*^ mutation to investigate the effects of the *mFicD*^*R371S*^ on pancreatic health and function. We observe that preventing the FicD-mediated deAMPylation of BiP compromises UPR in the pancreas and liver. Mutant *mFicD*^*R371S/R371S*^ mice exhibit signature markers for syndromic diabetes including glucose intolerance and reduced serum insulin levels. We also observe reduced β-cell function that progresses with age and is consistent with infant onset diabetes. Our pre-clinical murine model of *mFicD*^*R371S*^ serves as a valuable tool for investigating the pathology of this UPR related disease, providing critical insights into its underlying mechanisms.

## 2 Results

### 2.1 Generation of a mFicD^R371S^ mouse model

We utilized CRISPR-Cas9 homology-directed repair in C57Bl/6 zygotes to introduce the *mFicD*^*R371S*^ mutation in mice (Figure 1, Supplementary Fig. 1). From these founders, we established a *mFicD*^*+/R371S*^ mouse line, which was used for subsequent experiments. *mFicD*^*R371S/R371S*^ mice were born at expected Mendelian ratios, appeared normal in body size, and displayed no overt health issues compared to their *mFicD*^*+/+*^ and *mFicD*^*+/R371S*^ littermates (Figure 1B–C). To assess whether the *mFicD*^*R371S*^ mutation recapitulates the diabetic phenotypes in human patients, we measured blood glucose and serum insulin levels of *mFicD*^*R371S/R371S*^ mice and their sibling controls fed on a normal diet. By 5 weeks of age, *mFicD*^*R371S/R371S*^ mice exhibited elevated blood glucose and reduced serum insulin levels but maintained normal weight (Supplementary Figs. 2A–C). Of note, the serum proinsulin to insulin ratio remained unchanged between *mFicD*^*+/+*^ and *mFicD*^*R371S/R371S*^ mice, indicating that insulin processing is not impaired (Supplementary Fig. 2D). Glucose tolerance tests (GTT) and insulin tolerance tests (ITT) revealed that *mFicD*^*R371S/R371S*^ mice were glucose intolerant but respond well to insulin (Figure 1D–E). These observations are consistent with the neonatal diabetes phenotypes described in human *hFicD*^*R371S/R371S*^ patients [16].

### 2.2 The mFicD^R371S^ mutation recessively alters global AMPylation of BiP

**Figure 2:**
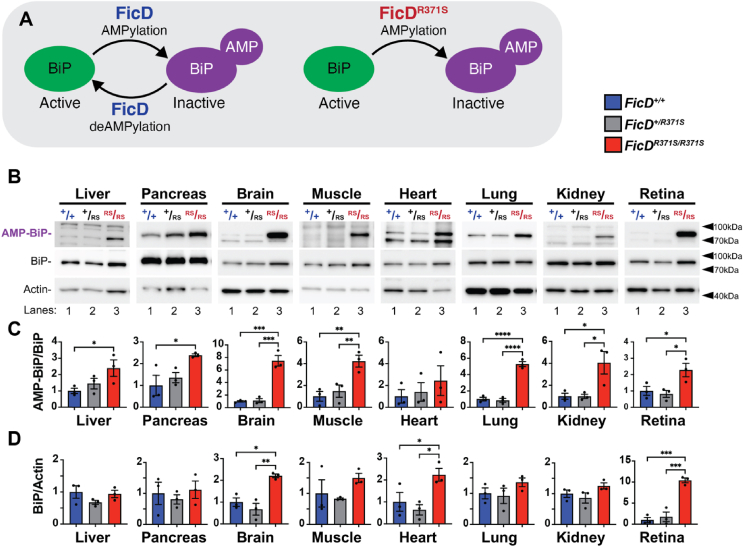
*The FicD*^*R371S*^*mutation recessively alters global AMPylation of BiP.* (A) Model of wildtype FicD and mutant FicD^R371S^ activity. (B) Representative Western blots of protein lysates isolated from *FicD*^*+/+*^, *FicD*^*+/R371S*^, and *FicD*^*R371S/R371S*^ tissues. Blots were probed with anti-AMP (17g6), anti-BiP, and anti-Actin antibodies. (C) Quantification of detected AMP-BiP (78 kDa) relative to detected BiP. (D) Quantification of BiP relative to Actin. N = 3. Bars indicate mean relative expression normalized to *FicD*^*+/+*^ controls, and error bars represent standard error. Statistics were performed using GraphPad Prism 10 using a 1-way ANOVA. ∗, p < 0.05; ∗∗, p < 0.01, ∗∗∗, p < 0.001.

Previous in vitro studies [16] have shown that FicD^R371*S*^ retains the ability to AMPylate BiP but lacks deAMPylation activity (Figure 2A). Since FicD is expressed ubiquitously, we hypothesized that BiP and BiP AMPylation levels would be elevated across various tissues in *mFicD*^*R371S/R371S*^ mice. Indeed, we observed significant increases in both total BiP protein and BiP AMPylation levels in multiple tissues of *mFicD*^*R371S/R371S*^ mice (Figure 2B–D). In contrast, AMPylation and BiP levels in *mFicD*^*+/R371*S^ and *m*FicD^+/+^ controls were indistinguishable, consistent with the recessive nature of hFicD^R371S^-induced neonatal diabetes in human patients.

To test how BiP AMPylation responds to pharmacologically induced ER stress, we administered a single intraperitoneal dose of tunicamycin (Tm, 1.0 mg/kg, dissolved in 150 mM dextrose) and sacrificed the mice 4 h post-injection. Control mice received vehicle injections. In response to Tm treatment, *mFicD*^*+/+*^, *mFicD*^*+/R371S*^, *mFicD*^*R371S/R371S*^ siblings all exhibited a marked reduction in BiP AMPylation in the liver. However, in *mFicD*^*R371S/R371S*^ liver, BiP AMPylation was unresponsive to tunicamycin (Supplementary Fig. 3A). Despite these alterations in BiP AMPylation/deAMPylation dynamics under stress conditions, there were no significant differences in UPR activation across genotypes in the liver following ER stress induction with tunicamycin (Supplementary Figs. 3B–G).

### 2.3 The mFicD^R371S/R371S^ mice exhibit reduced insulin secretion in response to glucose stimulation

**Figure 3:**
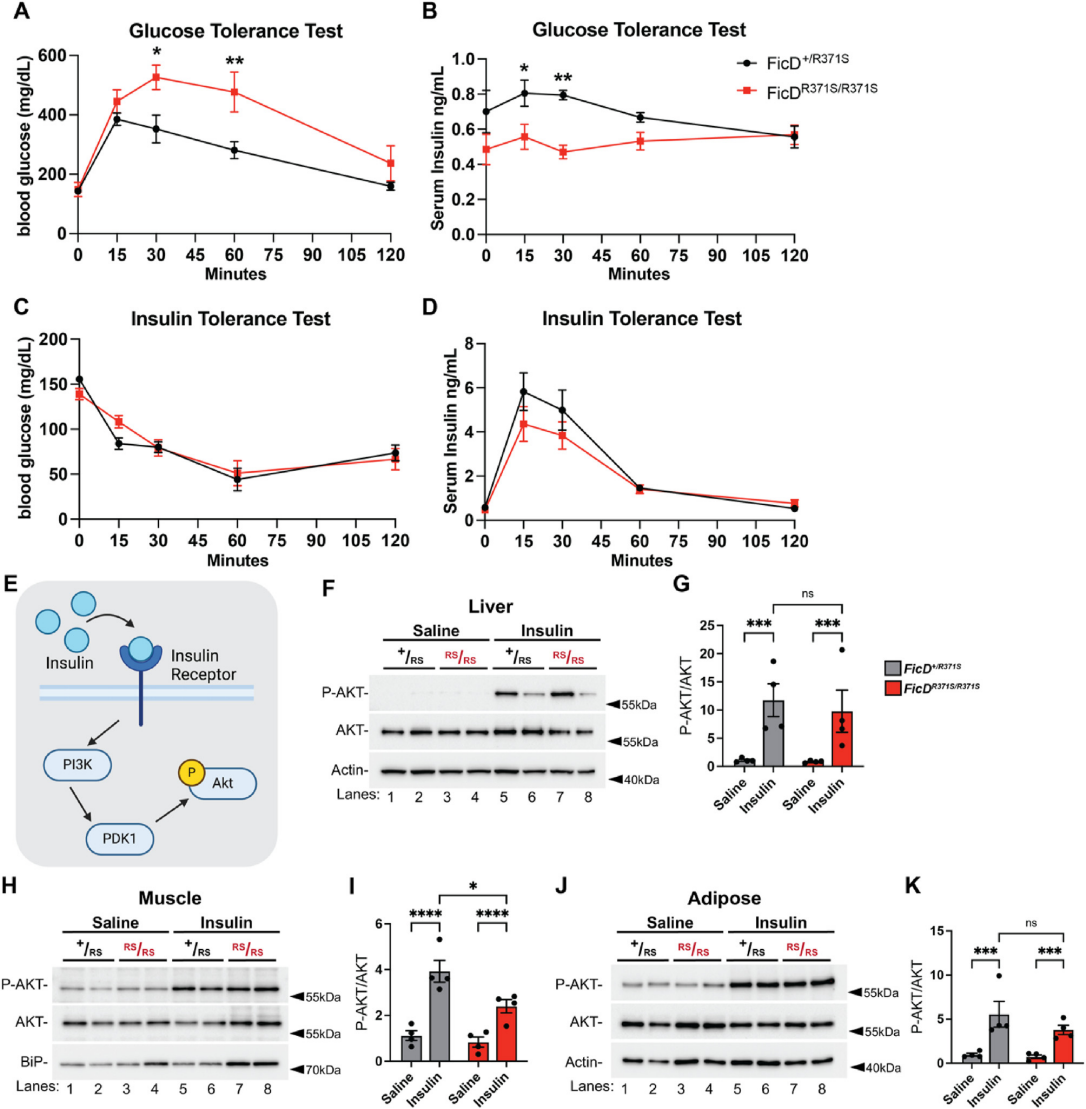
FicD^R371S^ mice respond normally to insulin. (A–B) GTT of 14- to 15-week-old female *FicD*^*+/R371S*^ (black) and *FicD*^*R371S/R371S*^ (red) mice N = 5–6. (C–D) ITT of 24-week-old female *FicD*^*+/R371S*^ (black) and *FicD*^*R371S/R371S*^ (red) mice N = 6. Both (A,C) blood glucose and (B,D) serum insulin levels were monitored. (E) Schematic model of Insulin stimulated AKT activation. Created in BioRender. Casey, A. (2025) https://BioRender. com/g98v964. (F–K) Representative Western blots and quantifications of protein lysates isolated from *FicD*^*+/R371S*^, and *FicD*^*R371S/R371S*^ (F–G) liver, (H–I) muscle, and (J–K) adipose tissue isolated from 10-week-old *FicD*^*+/R371S*^ (black) and *FicD*^*R371S/R371S*^ (red) mice treated with either saline or insulin. Blots were probed with anti-Phospho-AKT, anti-AKT, anti-BiP and anti-Actin antibodies. (G, I, K) Quantification of detected Phospho-AKT relative to detected AKT. Data indicate mean and error bars represent standard error. Statistics were performed using GraphPad Prism 10 using a 2-way ANOVA. ∗∗, p < 0.01; ∗∗∗, p < 0.001; ∗∗∗∗, p < 0.0001; ns, not significant.

Given that AMPylation of BiP is altered across multiple tissues, we considered the possibility that the diabetic phenotypes in *mFicD*^*R371S/R371S*^ mice might result from changes in insulin clearance or responsiveness. To investigate this, we performed GTT and ITT in female cohorts of *mFicD*^*+/R371S*^ and *mFicD*^*R371S/R371S*^ mice, measuring blood glucose and serum insulin levels in response to glucose and insulin administration (Figure 3A–D). Consistent with our previous observations, *mFicD*^*R371S/R371S*^ mice displayed glucose intolerance but remained responsive to insulin (Figure 3A,C). Upon glucose stimulation, heterozygotic *mFicD*^*+/R371S*^ controls exhibited a rise in serum insulin levels, whereas *mFicD*^*R371S/R371S*^ mice failed to increase insulin secretion (Figure 3B). However, following insulin injection, *mFicD*^*R371S/R371S*^ mice initially showed an increase in serum insulin levels, which then declined at a rate comparable to heterozygotic *mFicD*^*+/R371S*^ controls (Figure 3D). Taken together, these data confirms that *mFicD*^*R371S/R371S*^ is a recessive mutation and suggests that impaired insulin production, rather than altered insulin clearance, is the primary driver of the diabetic phenotypes in these mice.

### 2.4 Tissues in mFicD^R371S/R371S^ mice respond to insulin simulation

To determine whether ***mFicD*^*R371S/R371S*^** tissues remain responsive to insulin, we examined the activation of the PI3K/AKT signaling pathway, which is triggered when insulin binds to its receptor on the plasma membrane (Figure 3E) [18]. We treated *mFicD*^*+/R371*S^ and *m*FicD^*R371*S/*R371*S^ mice with either saline or insulin and performed Western blot analysis of liver, skeletal muscle, and adipose tissue (Figure 3F–K). Insulin stimulation increased AKT phosphorylation across all three tissues in both *mFicD*^*+/R371*S^ and *m*FicD^*R371*S/*R371*S^ mice (Figure 3F–K). Of note, there was no significant difference in AKT phosphorylation between *mFicD*^*+/R371*S^ and *m*FicD^*R371*S/*R371*S^ in liver or adipose tissue (Figure 3G,K). However, we observed a mild reduction in AKT phosphorylation in the skeletal muscle of *mFicD*^*R371S/R371S*^ mice (Figure 3H). These findings indicate that *mFicD*^*R371S/R371S*^ tissues remain responsive to insulin.

### 2.5 Loss of BiP deAMPylation alters UPR signaling in the pancreas and liver

**Figure 4:**
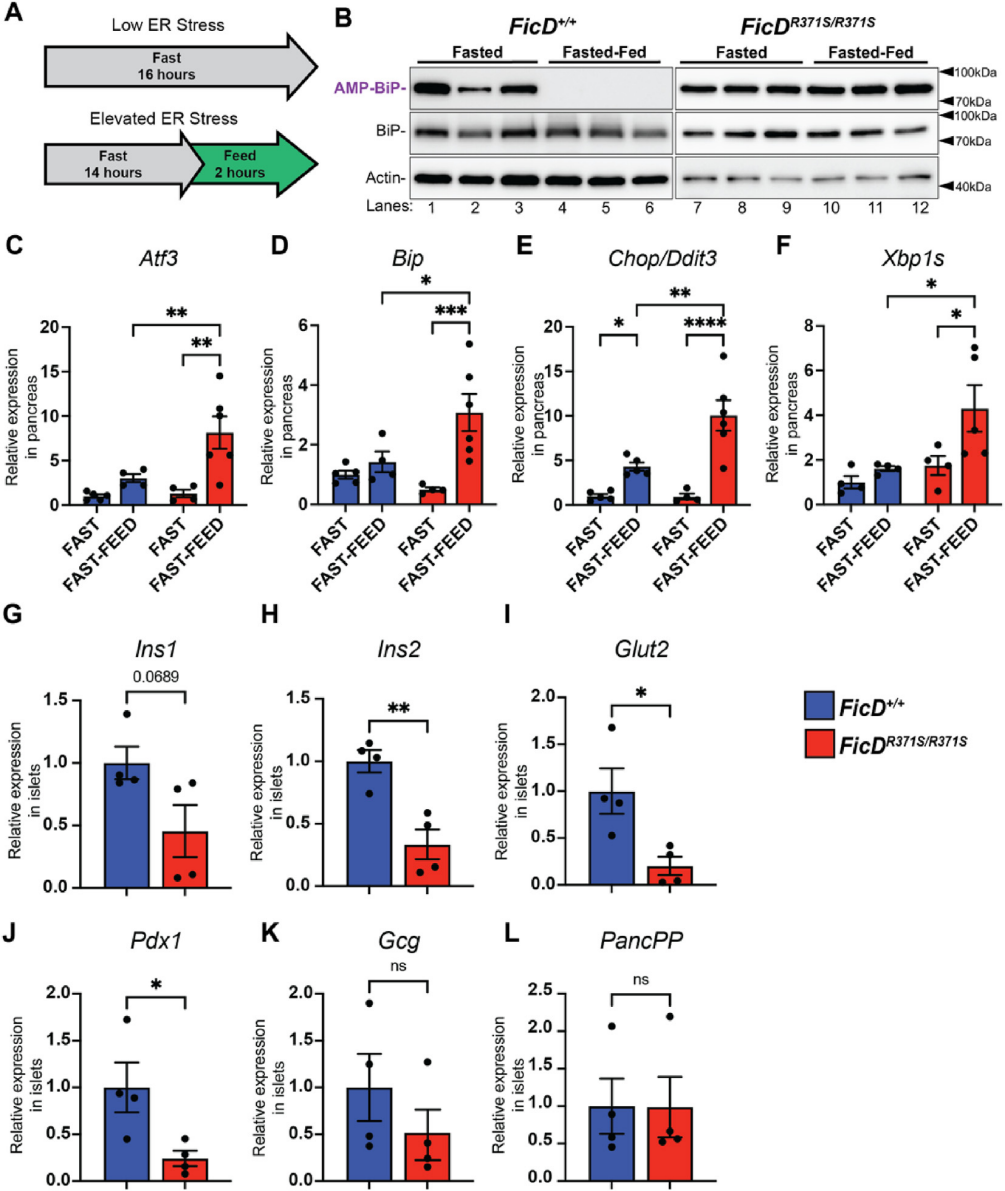
Loss of BiP deAMPylation alters UPR signaling in the pancreas and affects β-cell transcript expression. (A) A schematic representation of fasted, fasted-fed experimental conditions. (B) Representative Western blots of protein lysates isolated from *FicD*^*+/+*^ and *FicD*^*R371S/R371S*^ pancreas. Blots were probed with anti-AMP (17g6), anti-BiP, and anti-Actin antibodies. (C–F) *Atf3*, *BiP*, *Chop/Ddit3*, and *Xbp1s* mRNA analyzed by qPCR from *FicD*^*+/+*^ (blue bar) and *FicD*^*R371S/R371S*^ (red bar) mouse pancreas after fasting and fast-feeding. Expression values were normalized to *Gapdh*. Bars indicate mean relative expression compared to fasted *FicD*^*+/+*^ controls, and error bars represent standard error. N = 4–6. (G–L) Ins*1*, *Ins2*, *Glut2*, *Pdx1*, *Gcg*, and *PancPP* mRNA analyzed by qPCR from islets isolated from *FicD*^*+/+*^ (blue bar) and *FicD*^*R371S/R371S*^ (red bar) of 16–18-week-old male mice. Expression values were normalized to *Gapdh*. Bars indicate mean relative expression compared to *FicD*^*+/+*^ controls, and error bars represent standard error. N = 4–5. Statistics were performed using GraphPad Prism 10 using (C–F) 2-way ANOVA or (G–L) unpaired student's t-test. ∗, p < 0.05; ∗∗, p < 0.01; ∗∗∗, p < 0.001; ∗∗∗∗, p < 0.0001; ns, not significant.

Given the role of FicD-mediated BiP AMPylation/deAMPylation in ER stress regulation [8,19], we investigated whether UPR signaling was dysregulated in tissues of *mFicD*^*R371S/R371S*^ mice. We found that BiP AMPylation in pancreatic and liver lysates from *mFicD*^*+/+*^ mice, but not from *mFicD*^*R371S/R371S*^ mice, responded to mild physiological stress induced by fasting and refeeding (Figure 4A–B, Supplementary Fig. 4A). Additionally, UPR responsive genes were transcriptionally elevated in the pancreas and liver of *mFicD*^*R371S/R371S*^ mice compared to *mFicD*^*+/+*^ mice under fasting and refeeding conditions (Figure 3C–F Supplementary Figs. 4B–D), indicating dysregulation of UPR signaling in *mFicD*^*R371S/R371S*^ mice. Notably, *mFicD*^*R371S/R371S*^ mice exhibited a reduction in Glut2 transcript levels—the principal glucose transporter—in the liver during refeeding (Supplementary Fig. 4E). In contrast, the transcriptional repression of *Pck1*, a key enzyme in gluconeogenesis, remained consistent between *mFicD*^*+/+*^ and *mFicD*^*R371S/R371S*^ mice **(**Supplementary Fig. 4F).

Our findings show that *mFicD*^*R371S/R371S*^ mice exhibit elevated UPR signaling in response to fasting and refeeding (Figure 3, Supplementary Fig. 4), but not following tunicamycin induced UPR activation (Supplementary Fig. 3A). This pattern is consistent with our previous reports on FicD's role in modulating moderate physiological and environmental stresses [[8], [9], [10]]. The lack of a difference in response to acute pharmacological stress is likely due to the severe disruption of ER homeostasis caused by tunicamycin, which overwhelms the buffering capacity of FicD. As a result, cells experience stress beyond FicD's regulatory influence. These findings underscore a key distinction: FicD plays a specific role in adapting to physiological stress, whereas its influence is diminished under extreme pharmacological stress conditions.

### 2.6 β-cell transcript expression is altered in mFicD^R371S/R371S^ islets

To examine baseline ER stress in the endocrine pancreas before fasting, we isolated pancreatic islets from *mFicD*^*R371S/R371S*^ and *mFicD*^*+/+*^ sibling controls and performed qPCR to assess UPR signaling- and islet function-related transcripts (Figure 4G-L, Supplementary Fig. 5). Under non-stressed conditions, UPR transcript levels in *mFicD*^*R371S/R371S*^ islets were comparable to those in *FicD*^*+/+*^ controls, consistent with findings observed in the whole pancreas (Supplementary Fig. 5). However, analysis of endocrine cell-related transcripts revealed that many β-cell-specific transcripts were generally reduced in *mFicD*^*R371S/R371S*^ islets (Figure 3G–J). Notably, transcript levels for *Ins1* and *Ins2* (encoding insulin), *Glut2* (Glucose transporter type 2), and *Pdx1*(Pancreatic and duodenal homeobox 1) were reduced in *mFicD*^*R371S/R371S*^ islets. In contrast, the α-cell (Glucagon, *Gcg*) and F-cell (Pancreatic polypeptide, *PancPP*), appeared unaffected (Figure 4K-L).

### 2.7 Pancreatic islet organization and composition are disrupted in mFicD^R371S/R371S^ mice

Glucose intolerance, reduced insulin secretion, and downregulation of β-cell transcripts are hallmarks of declining β-cell health and function. To assess pancreatic tissue health in *mFicD*^*R371S/R371S*^ mice and their sibling controls, we performed Hematoxylin and Eosin (H&E) and TUNEL staining on paraffin embedded pancreas samples (Supplementary Fig. 6). Histological analysis revealed no signs of inflammation or fibrosis in the islets (Supplementary Fig. 5A), and there was no significant increase in TUNEL-positive cells, indicating a lack of significant apoptosis (Supplementary Fig. 5B).

**Figure 5:**
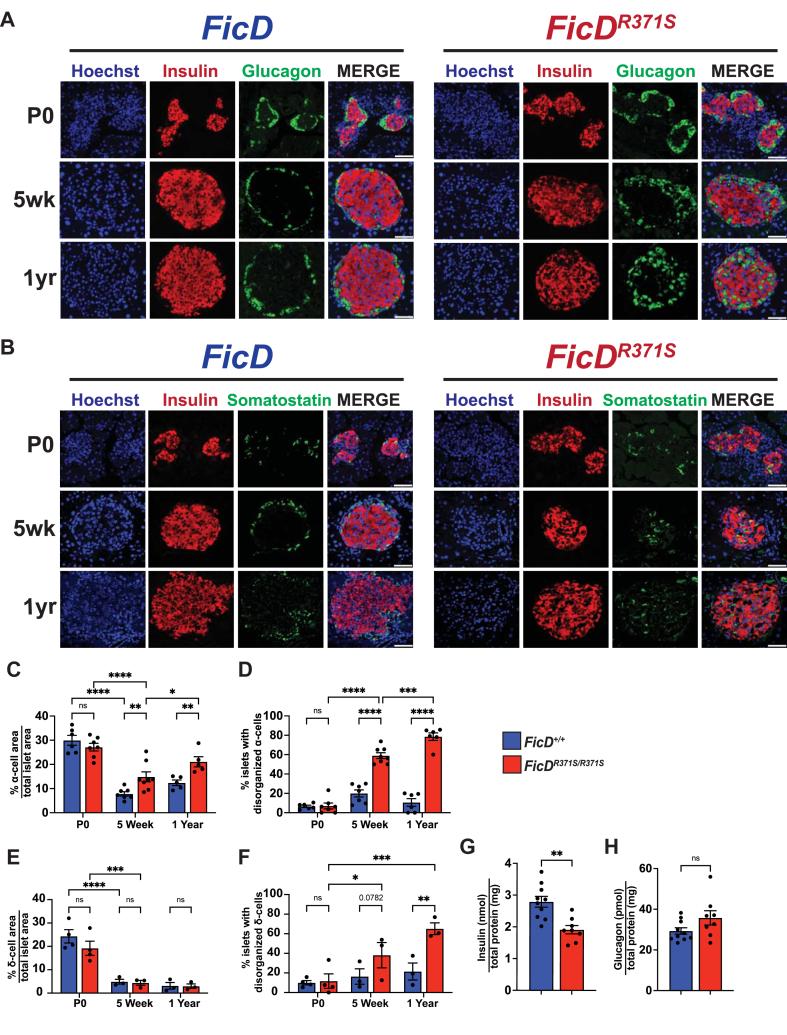
Regulation of FicD is required to maintain pancreatic islet organization and function. (A–B) Representative immunofluorescence images for (A) insulin and glucagon or (B) insulin and somatostatin expression in P0, 5-week, and 1-year-old *FicD*^*+/+*^ and *FicD*^*R371S/R371S*^ mice. Scale bar, 50uM. (C) Quantification of α-cells positive area as a percentage of total islet area of P0, 5-week, and 1-year-old *FicD*^*+/+*^ (blue bar) and *FicD*^*R371S/R371S*^ (red bar) mice. N = 6–8. (D) Quantification of percent of islets with disorganized (internal to islet) α-cells of P0, 5-week, and 1-year-old *FicD*^*+/+*^ (blue bar) and *FicD*^*R371S/R371S*^ (red bar) mice. N = 6–8. (E) Quantification of δ-cells positive area as a percentage of total islet area of P0, 5-week, and 1-year-old *FicD*^*+/+*^ (blue bar) and *FicD*^*R371S/R371S*^ (red bar) mice. N = 6–8. (F) Quantification of percent of islets with disorganized (internal to islet) δ -cells of P0, 5-week, and 1-year-old *FicD*^*+/+*^ (blue bar) and *FicD*^*R371S/R371S*^ (red bar) mice. N = 3. (G–H) Quantification of tissue levels of insulin and glucagon from 1-year-old *FicD*^*+/+*^ (blue bar) and *FicD*^*R371S/R371S*^ (red bar) pancreas. N = 8–10. Bars indicate mean, and error bars represent standard error. Statistics were performed using GraphPad Prism 10 using (C–F) 2-way ANOVA or (G–H) unpaired student's t-test. ∗, p < 0.05; ∗∗, p < 0.01; ∗∗∗, p < 0.001; ∗∗∗∗, p < 0.0001; ns, not significant. student's t-test. ∗, p < 0.05; ∗∗∗, p < 0.001, ns, not significant.

To further examine the organization and health of endocrine tissue, we performed immunostaining for insulin (β-cells) and glucagon (α-cells) or insulin (β-cells) and somatostatin (δ-cells) on paraffin embedded pancreas sections from *mFicD*^*+/+*^ and *mFicD*^*R371S/R371S*^ siblings (Figure 5A–B, Supplementary Fig. 7A). Islet organization was evaluated at multiple timepoints (P0, 5 weeks, 14 weeks and 1 year) to assess potential changes in islet structure over time (Figure 5C–F, Supplementary Figs. 7B–F).

At birth (P0), the islets in *mFicD*^*R371S/R371S*^ pancreases were well organized and indistinguishable from those in *mFicD*^*+/+*^ controls (Figure 5, Supplementary Fig. 7). However, by 5 weeks of age, *mFicD*^*R371S/R371S*^ islets contained a greater number of glucagon-positive α-cells compared to controls (Figure 5C). Moreover, the α-cells in *mFicD*^*R371S/R371S*^ mice were more disorganized, frequently scattered throughout the islet mass (Figure 5D). The disorganization of α-cell worsened with age, becoming more pronounced in 14-week-old and 1-year-old *mFicD*^*R371S/R371S*^ mice (Figure 5C, Supplementary Figs. 7B–C). A similar pattern of disorganization was observed in somatostatin-positive δ-cells within *mFicD*^*R371S/R371S*^ islets (Figure 5F, Supplementary Fig. 7D). Despite the structural disruption, the number of δ-cells in *mFicD*^*R371S/R371S*^ islets remained comparable to *mFicD*^*+/+*^ controls across all age groups tested (Figure 5E, Supplementary Fig. 7E). These findings suggests that disorganization of the α-cells and δ-cells and changes in cell type population represent distinct phenotypes. Furthermore, whereas the total amount of insulin in the *mFicD*^*R371S/R371S*^ pancreas appears reduced, there is no significant increase in pancreatic glucagon in 1-year-mice (Figure 5G–H). Despite these changes in cell composition and organization, the size of the pancreatic islets in *mFicD*^*R371S/R371S*^ mice did not significantly change with age (Supplementary Fig. 7F).

## 3 Discussion

### 3.1 A preclinical mouse model of neonatal diabetes and UPR dysregulation

In this study, we developed a preclinical mouse model for neonatal diabetes based on the hFicD^R371S^ mutation identified in humans, which disrupts FicD-mediated deAMPylation of BiP, impairing its regulatory function. Although *mFicD*^*R371S/R371S*^ mice appear healthy overall, they exhibit signs of insulin-dependent diabetes, characterized by glucose intolerance and impaired insulin secretion. While the islets of *mFicD*^*R371S/R371S*^ mice are normally organized at birth, they become progressively disorganized with age, accompanied by altered β-cell transcript expression and elevated UPR signaling in response to fasting-refeeding stress. Furthermore, these mice show defects in glucose stimulated insulin secretion, pointing to a primary defect in β-cell function rather than insulin turnover or peripheral insulin response.

Our data suggest that pancreatic dysfunction in *mFicD*^*R371S/R37S*^ mice is progressive, likely due to cumulative stress-induced damage. Since the pancreas appears to develop normally in utero, when blood glucose levels are regulated by the mother, we postulate that postnatal metabolic stress, such as feeding, exacerbates β-cell dysfunction over time.

In humans, homozygous *hFicD*^*R371S*^ mutations lead to multisystem disorders, including infancy-onset diabetes, severe neurodevelopmental delays, short stature, and cataracts. Similarly, *mFicD*^*R371S/R371S*^ mice exhibited elevated BiP AMPylation across multiple tissues, suggesting global BiP dysregulation. However, despite these widespread molecular changes, *mFicD*^*R371S/R371S*^ mice develop normally and show no overt health defects beyond progressive β-cell dysfunction. Many monogenetic mouse models for human diseases only partially recapitulate human disease [20,21], likely due to species-specific differences in metabolism, signaling, and lifespan. Because FicD regulates UPR signaling though BiP, additional physiological or chronic stressors may reveal phenotypes in other affected tissues, such as growth defects, neurodevelopmental delays, and cataracts seen in human patients [16,17].

### 3.2 Comparisons with other UPR-related diabetes models

UPR dysfunction is known contributor to *β*-cell failure in diabetes. For example, *PERK* mutations in humans cause Wolcott-Rallison syndrome, characterized by early onset diabetes and skeletal dysplasia, and osteopenia. *Perk*^*−/−*^ mice display phenotypes consistent with Wolcott-Rollison syndrome, including diabetes, skeletal abnormalities, and growth defects [22]. This model is distinct from our observations in *mFicD*^*R371S*^ mice as *Perk*^*−/−*^ mice display widespread apoptosis in the pancreas by 11 days old, including both endocrine and exocrine tissue [22].

In mice, *β*-cell specific deletions of the UPR sensor *Ire1α* (*Ire1α*^*β−/−*^) develop insulin dependent diabetes and exhibit defects in insulin processing [23]. Similarly, *β*-cell deletion of Xbp1, the major target of IRE1 endonuclease activity, also results in diabetic phenotypes in mice caused by defects in insulin processing and secretion [24]. Previous reports indicate that the size and number of islets is not altered in *Ire1α*^*β−/−*^ [23,25] as seen in our studies of *mFicD*^*R371S*^ mice (Supplementary Fig. 7F).

Of note, a recent study found contradictory phenotypes in *Ire1α*^*−/−*^ in islets when placed in the context of a genetic type I diabetes mouse model. These studies found that when backcrossed into non-obese diabetic (NOD) mouse models, *Ire1α*^*β−/−*^ islets exhibit increased numbers of ratios α-cell and δ-cell populations at 5 weeks of age [12], similar to our findings in *mFicD*^*R371S*^ mice (Figure 5). These phenotypes seen in *NOD Ire1α*^*β−/−*^ are similar to what we observe in *mFicD*^*R371S*^ mice. However, key differences remain between these two models. Unlike our observations that *mFicD*^*R371S*^ mice islet organization involves population changes in both α-cells and δ-cells and becomes more disordered with age, *NOD Ire1α*^*β−/−*^ islets appear to exhibit only a transient defects in insulin secretion and islet disorganization, which recovers over time and is protective from insulitis and the development of type I diabetes [12].

### 3.3 Relevance to human disease

FicD-mediated BiP AMPylation and deAMPylation is an evolutionarily conserved mechanism that modulates UPR signaling, allowing cells to adapt to physiological stress. Our findings suggest that disrupting FicD function leads to progressive β-cell dysfunction, not due to apoptosis, but through cumulative stress-related loss of plasticity. Recent studies have hypothesized that repetitive episodes of deleterious ER stress without proper recovery can enhance the progression of diabetes through loss of β-cell plasticity and function without an increase apoptosis [11]. Furthermore, this UPR induced *β*-cell dysfunction appears at least in some cases to be reversible. It is tempting to speculate that disruptions in both the *NOD Ire1α*^*β−/−*^ [12] and *mFicD*^*R371S*^ mouse models may stem from repetitive physiological ER stress. This is also consistent with findings in *ATF6α*^*−/−*^ mice which show no apparent signs of *β*-cell dysfunction under normal conditions but develop insulin deficiency when stressed with diet or combined with insulin secretion [26]. Understanding the role of IRE1 and ATF6 signaling in β-cell health and assessing whether attenuating UPR signaling could reverse β-cell dysfunction in *mFicD*^*R371S*^ mice will be a critical question moving forward.

## 4 Conclusion

Unlike other diabetes models that target specific UPR pathways or insulin processing, *mFicD*^*R371S*^ mice represent a model where global ER stress regulation is impaired. This disrupts adaptive stress responses across multiple tissues, making it a valuable tool for studying ER homeostasis in cells with high secretory demands, such as pancreatic β-cells. Understanding how FicD dysfunction contributes to β-cell failure may uncover novel therapeutic approaches for ER stress-related diabetes and metabolic diseases.

## 5 Methods

### 5.1 Mice

The Institutional Animal Care and Use Committee of the University of Texas Southwestern Medical Center approved all experiments. C57BL/6J and BDF1 mice were purchased from The Jackson Laboratory. CD-1 (ICR) mice were purchased from Envigo (Harlen). Mice were housed in a specific pathogen-free facility with a 12 h light dark cycle and fed an ad libitum standard chow diet (#2016 Harlan Teklad), unless specified. Fasting and fasting feeding studies were performed as previously described with 6-8-week-old females [8]. Fasting was performed overnight, with 2 h feeding occurring at 8am. Administration of 1.0 mg/kg of tunicamycin was performed as previously described [27]. Briefly, 8-10-week-old male mice were administered a single intraperitoneal dose (1.0 mg/kg) of tunicamycin (Sigma) dissolved in 150 mM dextrose at approximately 10am and sacrificed after 4 h. Mice in the control group were injected with vehicle alone. For insulin stimulation studies, mice were fasted for 5 h with water provided ad libitum starting at 8 am on the experimental day. Mice were administered a single intraperitoneal dose of (1U/kg) dose Insulin (Humalin) and sacrificed after 15 min. Mice in the control group were injected with saline alone.

### 5.2 Cas9 mRNA in vitro transcription

We modified the PX458 plasmid (Addgene plasmid # 48138) by adding T7 promoter upstream of Cas9 coding sequence and removing T2A-GFP. The modified plasmid was linearized by Not I (NEB) digestion. The linearized Cas9 plasmid and PCR products were purified using QIAquick PCR Purification Kit (QIAGEN). Cas9 mRNA was in vitro transcribed using purified linearized plasmid as a template by mMESSAGE mMACHINE™ T7 Transcription Kit (Invitrogen). Prepared Cas9 mRNA was then purified by Lithium chloride precipitation and dissolved in water for embryo transfer (Sigma: W1503).

### 5.3 Design sgRNA and donor repair template

The single guide RNAs (sgRNAs) and single-stranded repair template were design as described in [28]. Briefly DNA sequences where imported intro benching (https://benchling.com, 2022), and sgRNAs that cut near (<10bp) to the intended mutation site were designed with the Benchling design tool. sgRNAs were filtered by the folding quality using the WU-CRISPR server (https://crisprdb.org/wu-crispr-website/). The gRNA sequence (5’CTTTCATCGACGGCAACGG’3) was predicted to cut approximately 3 bp from our targeted mutation site and sgRNA was purchased from Synthego. The single-stranded oligodeoxynucleotide (ssODN) repair template, complementary to the PAM distal strand with 5′ and 3′ phosphorothioate ends to prevent exonuclease degradation and enhance HDR repair efficiency was purchased from IDT. (5'gaggacgccatgaacctgcacccagtcgagttcgcggccctggcccattacaaactggtgtacatccaccctttcatcgacggcaacggATCCacctcccgtctgctgatgaacctgattttgatgcaggcgggataccc’3), were the R370S mutation disrupts the PAM sequence and contains a silent mutation in G369 (GGG > GGA) to create a new BamHI site.

### 5.4 Embryo collection

Harvesting embryos was performed as described previously with slight modification [29,30]. Briefly, BDF1 female mice (4 weeks) were superovulated by intraperitoneal (IP) injection with 7.5 IU of PMSG (Prospec: #HOR-272), followed by IP injection of 7.5 IU of hCG (Sigma: #C1063) 48 later. After mating with C57BL/6J male mice, zygotes were harvested at E0.5 (the presence of a virginal plug was defined as embryonic day 0.5 (E0.5)) in mKSOM-Hepes from oviducts and cumulus cells were removed with hyaluronidase (Sigma: #H4272). Zygotes were cultured in the mKSOMaa until Cas9 mRNA/sgRNA/donor ssODN microinjection in a humidified atmosphere containing 5% (v/v) CO_2_ at 37 °C.

### 5.5 Microinjection of Cas9 mixture to zygotes

Microinjection of Cas9 mRNA/sgRNA/donor ssODN was performed as described previously [29] with slight modification. Briefly, the zygotes show clear two pronuclei were selected and transferred into a 40 μL drop of KSOM-Hepes and placed on an inverted microscope (Nikon, Japan) fitted with micromanipulators (Narishige, Japan). Mixture of Cas9 mRNA (100 ng/μL), sgRNA (50 ng/μL) and donor ssODN (20 ng/μL) was loaded to a blunt-end micropipette (Sutter Instrument, CA) of 2–3 μm internal diameter, and Piezo Micro Manipulator (Prime Tech Ltd, Japan) was used to create a hole in the zona pellucida and the zygote membranes. The injection of the Cas9 mixture into the cytoplasm of zygotes was confirmed by the bulge of membrane. Groups of 12 zygotes were manipulated simultaneously, and each session was limited to 10 min. After microinjection, the zygotes were cultured in the 40 μL droplet of mKSOMaa for 3 days in a humidified atmosphere of 5% CO_2_, 5% O_2_ in air at 37 °C.

### 5.6 Embryo transfer

Embryo transfer was performed as described previously [29,30]. Briefly, CD-1 female mice (8 weeks old or older) were mated with vasectomized CD-1 male mice to induce pseudopregnancy. 8–13 embryos at E3.5 were surgically transferred to the surrogate uterine at E2.5 under anesthesia with Ketamine (30 mg/mL)/Xylazine (4 mg/mL) and analgesia with Buprenorphine SR-LAB (1 mg/mL) within 20–30 min per surrogate. F0 mice were bred with C57Bl/6J mice to obtain F1 mice heterozygous for the FicD^R371S^ mutation.

### 5.7 Genotyping and DNA sequencing

To determine genotypes of full-term delivered pups, tail-tips were used for genomic DNA extraction using DNeasy Blood and Tissue kit (Qiagen). The genomic DNA sequences including target site were amplified with PrimeSTAR GXL DNA Polymerase. PCR products were purified with QIAquick PCR Purification kit (Qiagen) and submitted for Sanger sequencing (Eurofins Genomics) to confirm the FicD^R371S^ mutation. Forward primer: 5′ tcctgcacgccctcaagatgga 3′ Reverse primer: 5′ cgtcaccctcgttggcgacttc 3’. All mice used in this study were genotyped via PCR using the primer set (5′ gggggtggttcaaggaag 3′ and downstream 5′ ctgcaacctcctactggc 3’ followed by restriction digestion with BamH1). Amplicons of total FicD gene were submitted for Sanger sequencing to confirm the FicD^R371S^ mutation. F0 mice were bred with C57Bl/6J mice to obtain F1 mice heterozygous for the FicD^R371S^ mutation.

### 5.8 Glucose tolerance test and insulin tolerance test

For glucose tolerance tests (GTTs) and insulin tolerance tests (ITTs), mice were fasted for 6 h with water provided ad libitum starting at 7–8 am on the experimental day. Baseline glucose levels were collected 1 h prior to GTT or ITT. During GTT or ITT, blood glucose levels were monitored at 0, 15, 30, 60, 90, and 120 min after an intraperitoneal injection of 2.0 g/kg body weight dose of glucose (Gibco 20% ref A24940-01, lot 2562566) or 1U/kg body weight dose of Insulin (Humalin), respectively. Blood samples were collected from the tail vein, glucose was measured using a glucometer.

### 5.9 Islet isolations

Pancreatic islets were isolated from mice as previously described [31]. Briefly, the pancreas was digested via injection through the pancreatic duct with Collagenase in Hank's balanced salt solution. Digestion was halted through washes with Hank's balanced salt solution and islet/exocrine tissue was transferred to RPMI 1640. Islets were hand-picked before used in experiments.

### 5.10 Assessment of serum pancreatic insulin, proinsulin, and glucagon levels

Insulin and glucagon were extracted from the pancreas using the acid ethanol method [32]. For serum isolation, blood was collected in Microvette serum collection tubes (Sarstedt) and centrifuged at 10,000×*g* at 4 °C for 15 min. Insulin and Glucagon levels were measured using a commercially available ELISA kits (Crystal Chem., Inc.). Proinsulin levels were measured using a commercially available ELISA kits (Novus).

### 5.11 Histology and immunohistochemistry

Mouse pancreases were harvested and fixed in 10% neutral buffered formalin overnight at 4 °C. Paraffin sections were embedded by the UT Southwestern (UTSW) Histo Pathology Core. Hematoxylin and eosin (H&E) staining was performed using standard techniques [33]. Nuclei were visualized with Hoechst. TUNEL/PI staining was performed by the UTSW Histo Pathology Core using the DeadEnd™ Fluorometric TUNEL system (Promega). Pancreatic Glucagon, Insulin, and Somatostatin distribution was determined using deparaffinized pancreas section after a hot citric acid buffer (10 mM citric acid, 0.05% Tween-20, pH 6.0) antigen retrieval. Sections were incubated with anti-insulin (Abcam ab181547), anti-somatostatin (Santa Cruz Biotech, sc-74556), and anti-glucagon (Invitrogen 14-9743-82) antibodies at 4 °C for 16 h. Secondary antibodies used were Alexa Fluor 488 goat anti-mouse (Invitrogen) and Alexa Fluor 555 goat anti-rabbit (Invitrogen).

### 5.12 Quantitative real-time PCR

RNA from total pancreas and isolated islets was extracted using RNA Stat-60 (Iso-Tex Diagnostics). Complementary DNA (cDNA) was generated from RNA (2 μg) using the High-Capacity cDNA Reverse Transcription Kit (Life Technologies). qPCR was performed by the SYBR Green method [34]. DNA contamination in RNA and reagents was assessed using no reverse transcriptase samples in which qPCR reactions were performed on mock cDNA from samples prepared without reverse transcriptase and no template control samples in which no template was added to the qPCR reaction. Primer sequences for the genes analyzed can be found in Table S1. Experiments were performed on a BioRad CFX Touch and analyzed with CFX Maestro Software.

### 5.13 Western blot analysis

Collected tissues were washed in PBS and homogenized in RIPA buffer (50 mM Tris pH 8, 150 mM NaCl, 1% NP-40, 0.5% Na deoxycholate, PMSF, PhosSTOP (Roche), Protease Inhibitor Cocktail (Roche. Lysates were centrifuged at 10,000×*g* for 10 min to remove nuclei and cellular debris. Lysates were separated by SDS-PAGE and transferred to PVDF membranes. Blots were probed with anti-AMP 17g6 (gift from Aymelt Itzen) [35], anti-GRP78 (Abcam, ab21685), anti-AKT(Cell Signaling #9272), and anti-Phospho-AKT (Cell Signaling #mAB4058), and anti-actin (Sigma A2228). Membranes were then incubated with horseradish peroxidase–conjugated secondary antibodies Goat Anti-Mouse IgG-HRP (Abcam, ab205719) or Donkey anti-Rabbit IgG-HRP (Fisher, NA934) against the primary antibody's host species for 1 h. Membranes were developed using the ECL substrate solution (Bio-Rad). Quantification of western blots was performed using NIH ImageJ software. Band densities were measured and subtracted from background.

## CRediT authorship contribution statement

**Amanda K. Casey:** Writing – review & editing, Writing – original draft, Visualization, Supervision, Project administration, Investigation, Funding acquisition, Conceptualization. **Nathan M. Stewart:** Visualization, Investigation. **Naqi Zaidi:** Investigation. **Hillery F. Gray:** Investigation. **Hazel A. Fields:** Investigation. **Masahiro Sakurai:** Investigation. **Carlos A. Pinzon-Arteaga:** Investigation. **Bret M. Evers:** Writing – review & editing. **Jun Wu:** Writing – review & editing, Funding acquisition, Conceptualization. **Kim Orth:** Writing – review & editing, Writing – original draft, Supervision, Project administration, Funding acquisition, Conceptualization.

## Declaration of competing interest

The authors declare that they have no known competing financial interests or personal relationships that could have appeared to influence the work reported in this paper.

## Data Availability

Data will be made available on request.
