# Supplementary material for: Pre-clinical model of dysregulated FicD AMPylation causes diabetes by disrupting pancreatic endocrine homeostasis
Source: Mol Metab. 2025 Mar 10;95:102120. doi: 10.1016/j.molmet.2025.102120 (PMC11964657; doi:10.1016/j.molmet.2025.102120)
Supplement: Multimedia component 1 [file mmc1.docx]

**Supplementary Materials
for**

**Pre-Clinical Model of Dysregulated FicD AMPylation causes diabetes by disrupting pancreatic endocrine homeostasis**

Amanda K. Casey, Nathan M. Stewart, Naqi Zaidi, Hillery F. Gray, Hazel A. Fields, Masahiro Sakurai, Carlos A. Pinzon-Arteaga, Bret M. Evers, Jun Wu*, Kim Orth**

* Correspondence: [jun2.Wu@utsouthwestern.edu](mailto:jun2.Wu@utsouthwestern.edu)

** Correspondence: [kim.orth@utsouthwestern.edu](mailto:kim.orth@utsouthwestern.edu)

**Supplementary Figures 1–7 and Supplementary Table 1**

# Supplemental figures


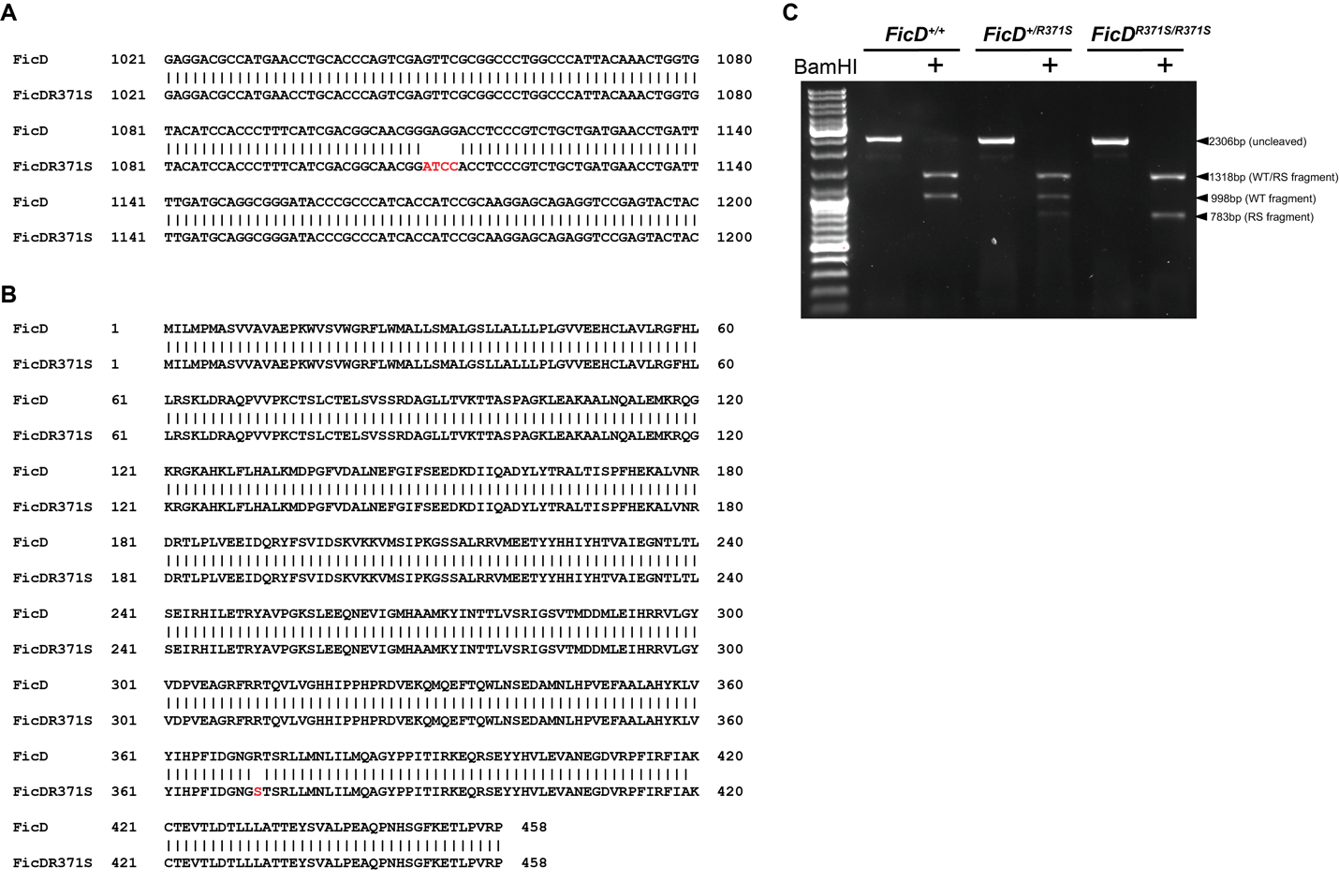


## Supplementary Figure 1. Generation of a neonatal diabetic FicD^R371S^ mouse model.

(A) The aligned predicted DNA sequences of *FicD and FicD^R371S^* from nt1021-1200 . FicD is identical to Fic*^R371S^* except for 4 nucleotide mutations that confer a single amino acid change and silent BamHI restriction site. (B) The aligned predicted protein sequences of FicD and FicD^R371S^. FicD is identical to Fic*^R371S^* except for R to S point mutation at residue 371. (C) Representative agarose gel of PCR amplicons and digests for FicD genotyping. Primers were used that amply across the modified region of *FicD*, producing a 2306bp *FicD* amplicon. Digestion of *FicD* amplicons with BamHI result in DNA fragments of 1318 and 988 bp (FicD), 1318 and 783 bp (FicD^R371S^).

##
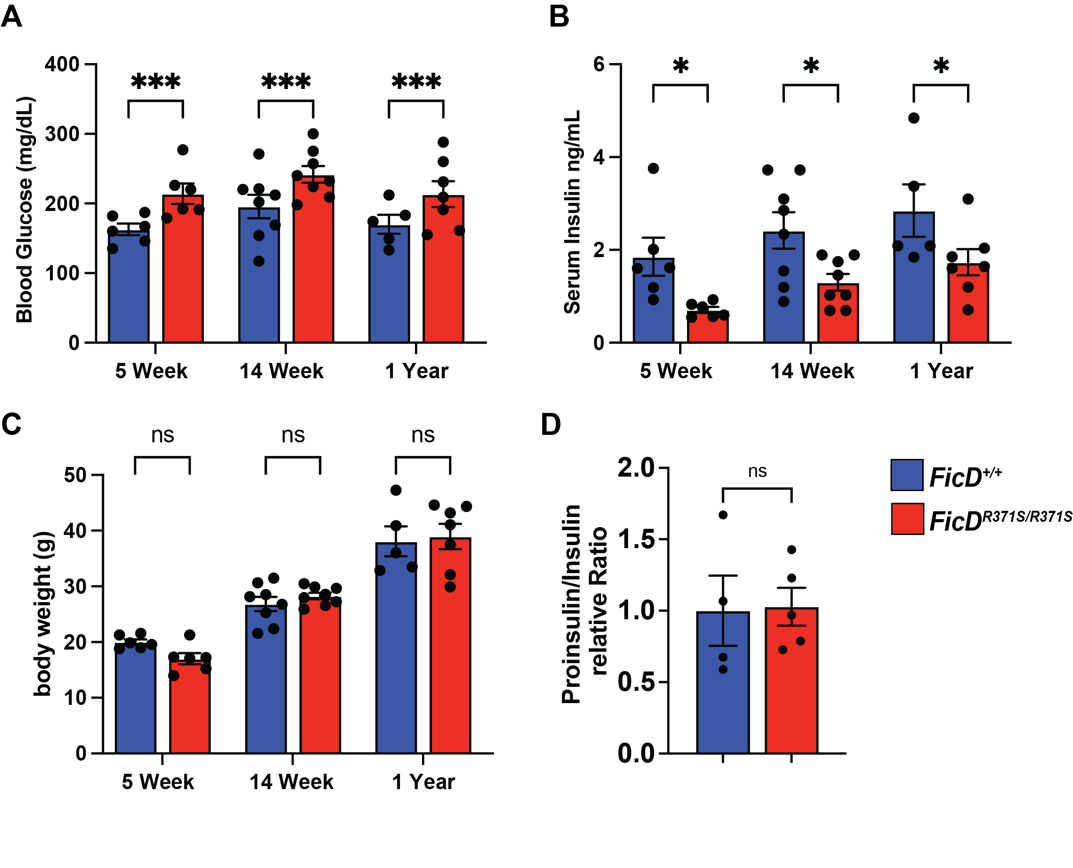


## Supplementary Figure 2. Initial characterization of a neonatal diabetic FicD^R371S^ mouse model.

(A-C) Quantification of (A)blood glucose levels, (B) serum insulin levels, and (C) body weights of 5-week, 14-week, and 1-year-old *FicD^+/+^* (blue bar) and *FicD^R371S/R371S^* (red bar) mice N=5-8. (D) Quantification of serum proinsulin to insulin ratios of 1-year-old *FicD^+/+^* (blue bar) and *FicD^R371S/R371S^* (red bar) mice relative to *FicD^+/+^* controls N=4-5. Bars indicate mean, and error bars represent standard error. Statistics were performed using GraphPad Prism 10 using (A-C) 2-way ANOVA or (D) unpaired student’s t-test. *, p < 0.05; *** , p <0.001; ns, not significant.


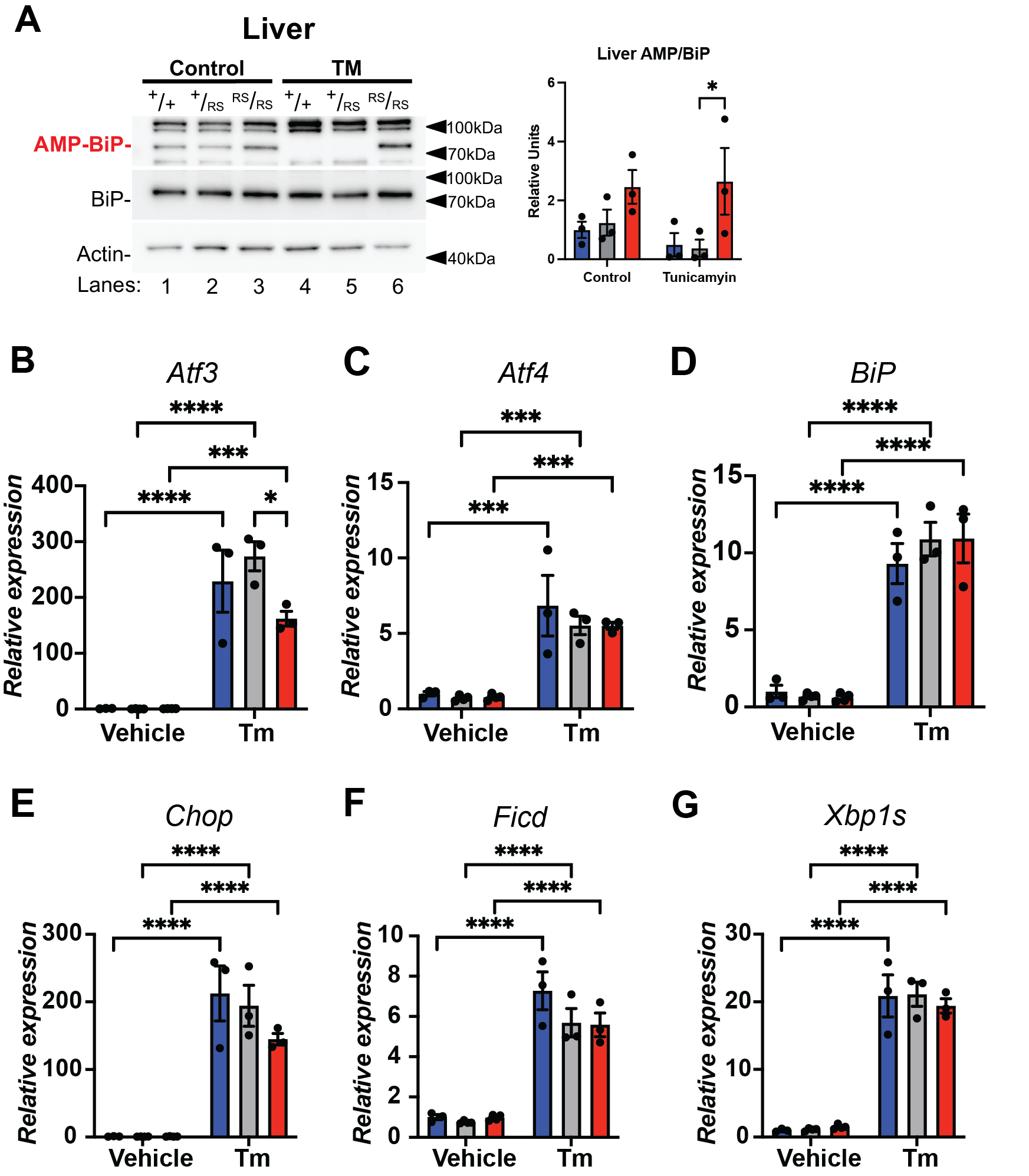


## Supplementary Figure 3. FicD^R371S^ mutation recessively alters AMPylation of BiP but not UPR signaling in liver.

(A) Representative Western blots of protein lysates isolated from *FicD^+/+^*, *FicD^+/R371S^*, and *FicD ^R371S/R371S^* liver treated with 1mg/kg Tunicamycin or control. Blots were probed with anti-AMP (17g6), anti-BiP, and anti-Actin antibodies. Quantification of detected AMP-BiP relative to detected BiP and normalized to *FicD^+/+^* control. (B-G) *Atf3*, *Atf4*, *BiP*, *Chop/Ddit3*, *Ficd*, and *Xbp1s* mRNA analyzed by qPCR from *FicD^+/+^* (blue bar), *FicD^+/R371S^* (gray bar), and *FicD^R371S/R371S^* (red bar) mouse pancreas after 4hrs administration of vehicle control or 1mg/kg Tunicamycin (Tm). Expression values were normalized to *Gapdh*. Bars indicate mean relative expression compared to vehicle *FicD^+/+^* controls, and error bars represent standard error. N=3. Statistics were performed using GraphPad Prism 10 using 2-way ANOVA *, p < 0.05; ** , p <0.01; *** , p <0.001; **** , p <0.0001; ns, not significant.

##
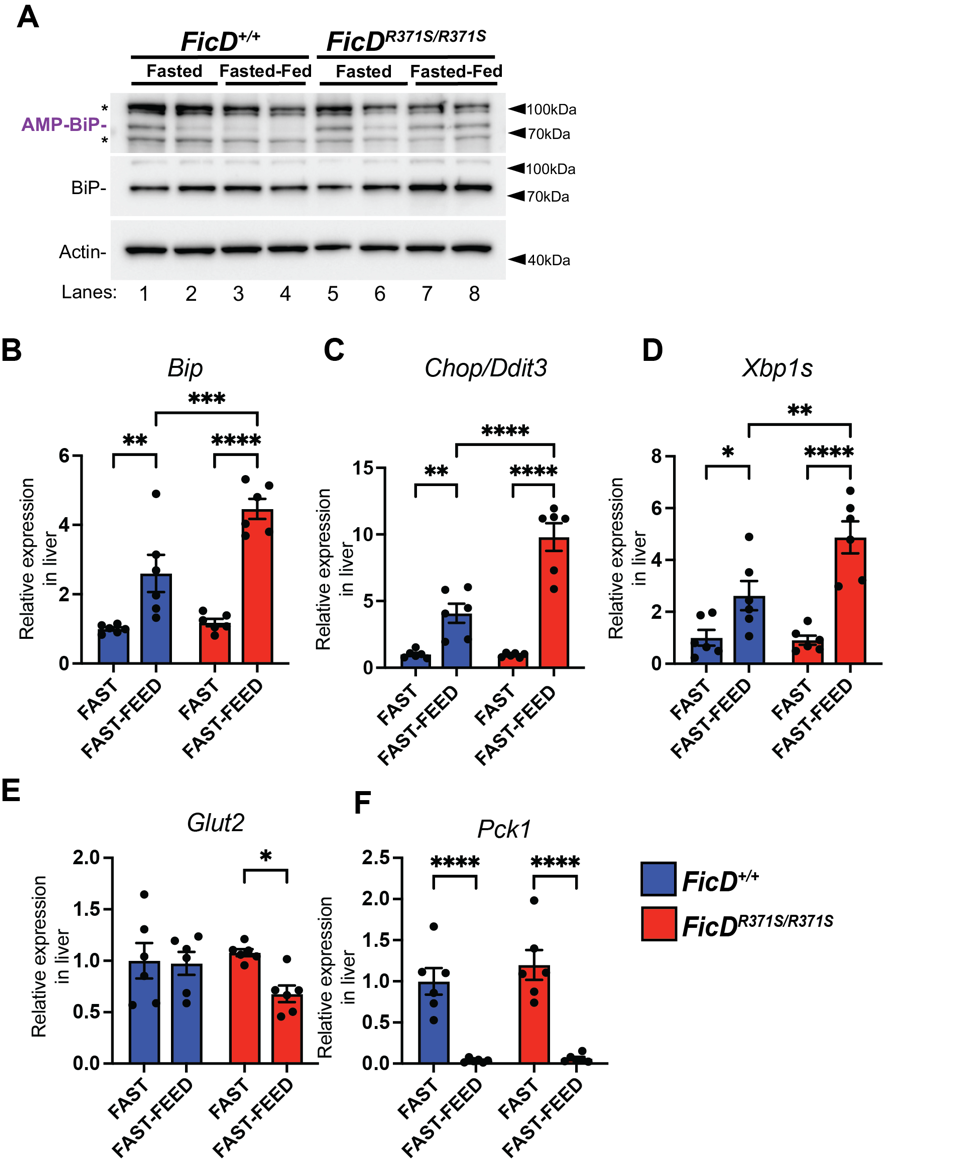


## Supplementary Figure 4. Loss of BiP deAMPylation alters UPR signaling in the liver and affects Glut2 transcript expression.

(A) Representative Western blots of protein lysates isolated from *FicD^+/+^* and *FicD ^R371S/R371S^* Liver. Blots were probed with anti-AMP (17g6), anti-BiP, and anti-Actin antibodies. (B-F) *BiP*, *Chop/Ddit3*, *Xbp1s, Glut2,* and *Pck1* mRNA analyzed by qPCR from *FicD^+/+^* (blue bar) and *FicD^R371S/R371S^* (red bar) mouse liver after fasting and fast-feeding. Expression values were normalized to *Gapdh*. Bars indicate mean relative expression compared to fasted *FicD^+/+^* controls, and error bars represent standard error. N=5-6. Statistics were performed using GraphPad Prism 10 using 2-way ANOVA. *, p < 0.05; ** , p <0.01; *** , p <0.001; **** , p <0.0001; ns, not significant.

*
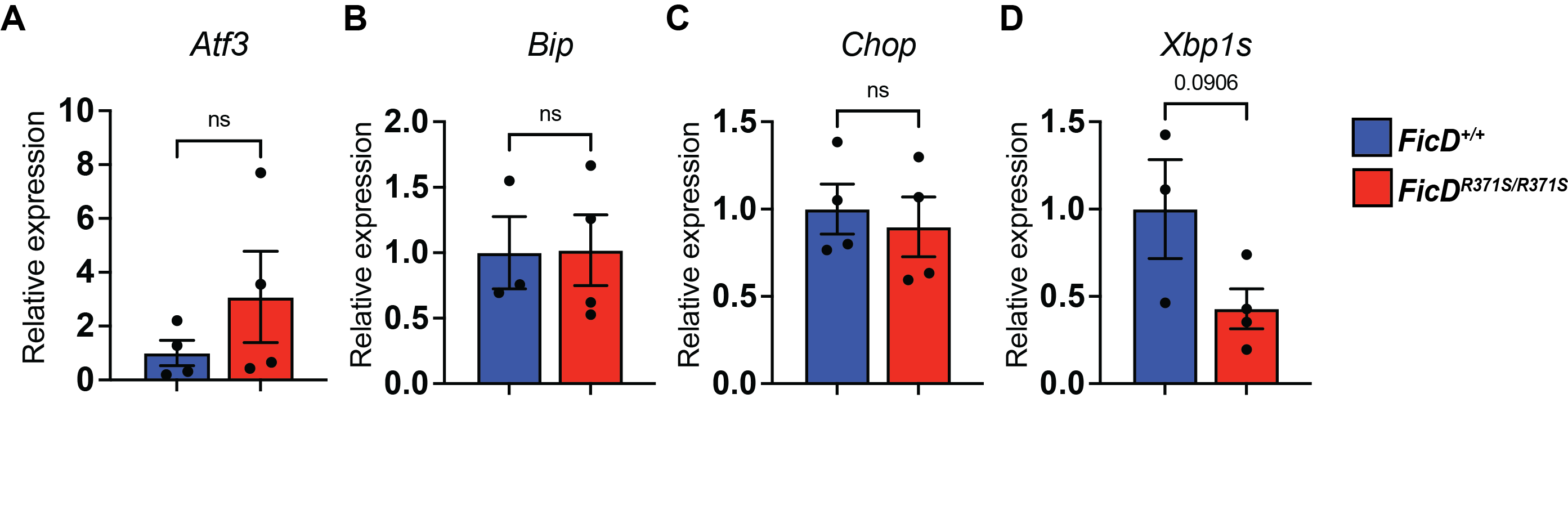
*

## Supplementary Figure 5. Loss of BiP deAMPylation does not alter baseline UPR transcript levels in β-cells.

(A-D) *Atf3*, *BiP*, *Chop/Ddit3*, and *Xbp1s* mRNA analyzed by qPCR islets isolated from *FicD^+/+^* (blue bar) and *FicD^R371S/R371S^* (red bar) mice. Expression values were normalized to *Gapdh*. Bars indicate mean relative expression compared to *FicD^+/+^* controls, and error bars represent standard error. N=4-5. Statistics were performed using GraphPad Prism 10 using unpaired student’s t-test. ns, not significant.


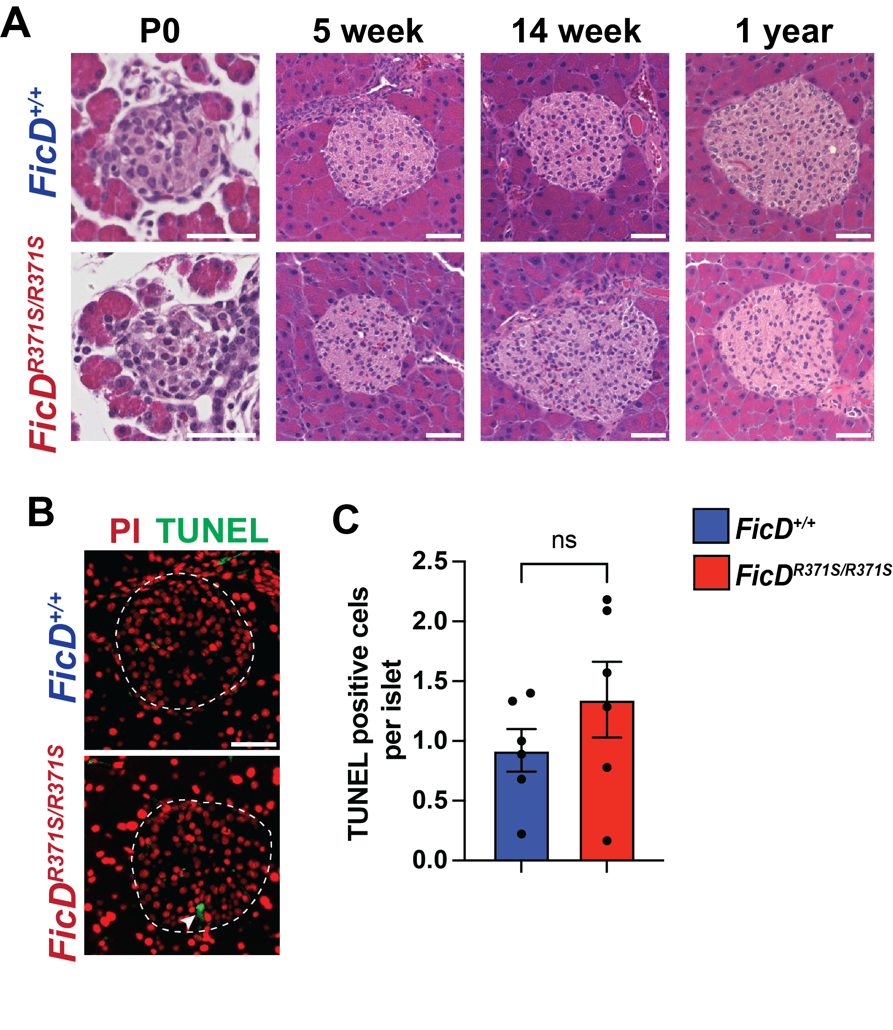


## Supplementary Figure 6. Regulation of FicD is not required to for pancreatic islet survival.

(A) Representative H&E (hematoxylin and eosin) in P0, 5-week, 14-week, and 1-year-old *FicD^+/+^* and *FicD^R371S/R371S^* mice. (B) Representative TUNEL (Terminal deoxynucleotidyl transferase dUTP nick end labeling) and PI (propidium iodide) stained images of pancreas of 5 week old *FicD^+/+^*(blue bar) and *FicD^R371S/R371S^* (red bar) mice. Scale bar, 50uM. (C) Quantification of percent TUNEL positive cells per islet of 5 week old *FicD^+/+^* and *FicD^R371S/R371S^* mice. Bars indicate mean, and error bars represent standard error. N=6. Statistics were performed using GraphPad Prism 10 using unpaired student’s t-test. ns, not significant.


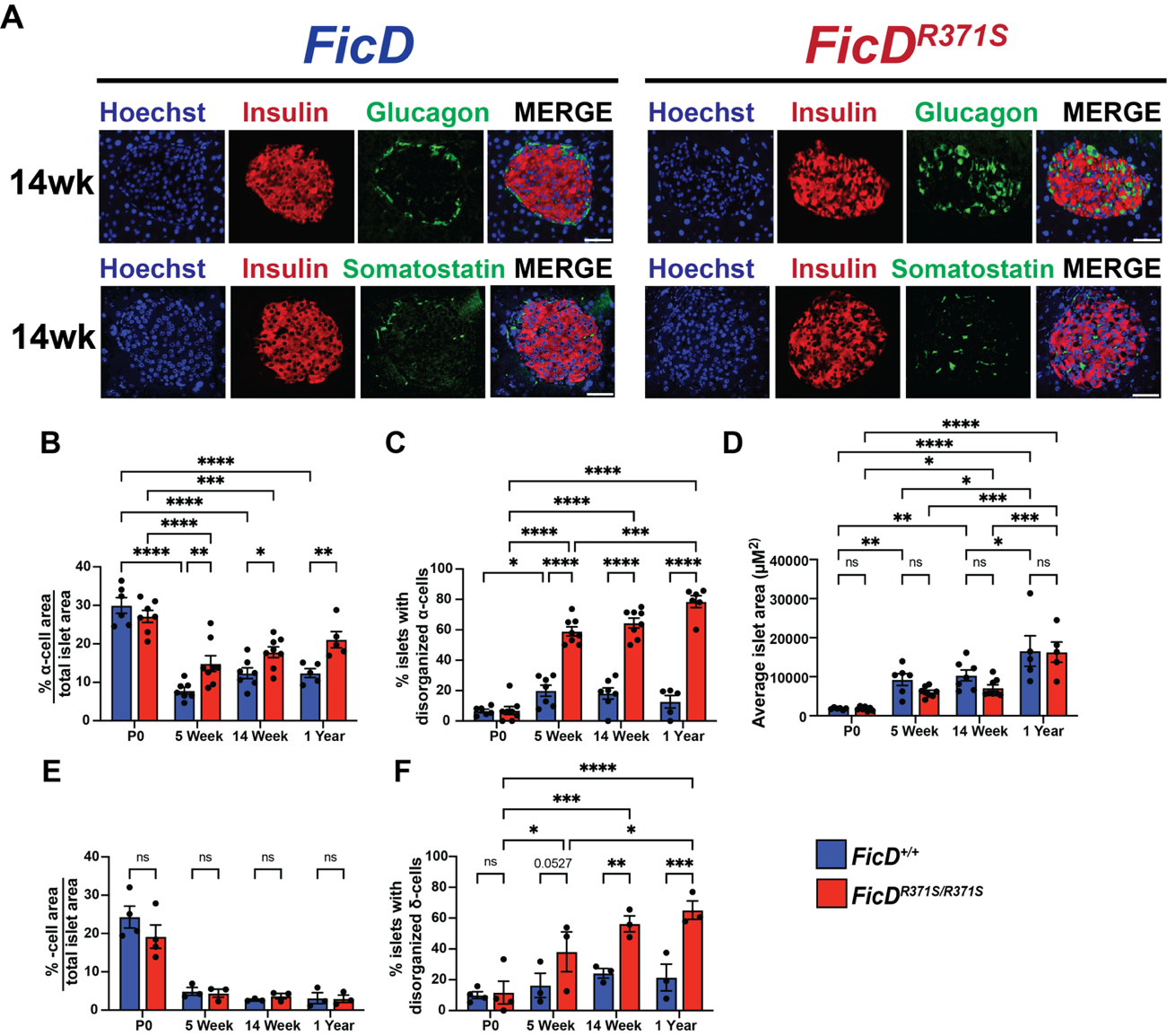


**Supplementary Figure 7. Regulation of FicD is required to maintain pancreatic islet organization and function.**

(**A**)Representative immunofluorescence images for insulin, glucagon, and somatostatin expression in 14 week old *FicD^+/+^* and *FicD^R371S/R371S^* mice. Scale bar, 50uM. (**B**) Quantification of α-cells positive area as a percentage of total islet area of P0, 5-week, 14-week, and 1-year-old *FicD^+/+^* (blue bar) and *FicD^R371S/R371S^* (red bar) mice. (**C**) Quantification of percent of islets with disorganized (internal to islet) α-cells of P0, 5-week, 14-week, and 1-year-old *FicD^+/+^* (blue bar) and *FicD^R371S/R371S^* (red bar) mice. (**D**) Quantification of percent of islets with disorganized (internal to islet) δ-cells of P0, 5-week, 14-week, and 1-year-old *FicD^+/+^* (blue bar) and *FicD^R371S/R371S^* (red bar) mice. (**E**) Quantification of δ-cells positive area as a percentage of total islet area of P0, 5-week, 14-week, and 1-year-old *FicD^+/+^* (blue bar) and *FicD^R371S/R371S^* (red bar) mice. (**F**) Quantification of islet area of P0, 5-week, 14-week, and 1-year-old *FicD^+/+^* (blue bar) and *FicD^R371S/R371S^* (red bar) mice. Bars indicate mean, and error bars represent standard error. N=6-8. Statistics were performed using GraphPad Prism 10 using 2-way ANOVA. *, p < 0.05; ** , p <0.01; *** , p <0.001; **** , p <0.0001; ns, not significant.

| **Gene name** | **Forward primer sequence** | **Reverse primer sequence** |
| --- | --- | --- |
| *Atf3* | 5’ TGGAGATGTCAGTCACCAAGTCT 3’ | 5’ GCAGCAGCAATTTTATTTCTTTCT 3’ |
| *Atf4* | 5’ ACTCTAATCCCTCCATGTGTAAAGG 3’ | 5’ CAGGTAGGACTCTGGGCTCAT 3’ |
| *BiP* | 5’ CAAGGATTGAAATTGAGTCCTTCTT 3’ | 5’ GGTCCATGTTCAGCTCTTCAAA 3’ |
| *Chop/Ddit3* | 5’ CCAGAAGGAAGTGCATCTTCA 3’ | 5’ ACTGCACGTGGACCAGGTT 3’ |
| *Ficd* | 5’ GTAGACGCACTGAATGAGTTCG 3’ | 5’ TGGTGTATAAGTAGTCAGCCTGG 3’ |
| *Gapdh* | 5’ AGGTCGGTGTGAACGGATTTG 3’ | 5’ TGTAGACCATGTAGTTGAGGTCA 3’ |
| *Glut2* | 5’ ACACCGGAATGTTCTTAGCC 3’ | 5’ GTGAGAAGCCGAGGAAAG 3’ |
| *Ins1* | 5’ GCCATGTTGAAACAATGACCT 3’ | 5’ CAGAGAGGAAGGTACTTTGGACTATAA 3’ |
| *Ins2* | 5’ GAAGTGGAGGACCCACAAGT 3’ | 5’ AGTGCCAAGGTCTGAAGGTC 3’ |
| *PancPP* | 5’ TCACTAGCTCAGCACACAGGA 3’ | 5’ CCACCCAAGTGGATACGAGA 3’ |
| *Pck1* | 5’ TTGAACTGACAGACTCGCCCT 3’ | 5’ TGCCCATCCGAGTCATGA 3’ |
| *Pdx1* | 5’ GAAATCCACCAAAGCTCACG 3’ | 5’ CGGGTTCCGCTGTGTAAG 3’ |
| *Gcg* | 5’ GCCCTTCAAGACACAGAGGA 3’ | 5’ CCTCATGCGCTTCTGTCTGv |
| *Xbp1S* | 5’CTGAGTCCGCAGCAGGT 3’ | 5’ TGTCAGAGTCCATGGGAAGA 3’ |
|  |  |  |

## Supplementary Table 1. Primer sequences for the genes analyzed in this study by qPCR.
